# Tuberculosis preventive treatment in newborns

**DOI:** 10.1016/j.jped.2024.06.009

**Published:** 2024-08-08

**Authors:** Tony TannousTahan, Andrea Maciel de Oliveira Rossoni, Giuliana Lugarini, Simoni Pimenta de Oliveira, Juliana Taques, Mauricio Bedim dos Santos, Betina Mendez Alcântara Gabardo, Tatiane Emi Hirose, Cristina de Oliveira Rodrigues

**Affiliations:** a Universidade Federal do Paraná, Curitiba, PR, Brazil; b Rede Brasileira de Pesquisa em Tuberculose – RedeTB, Brazil; c Universidade Tecnológica Federal do Paraná, Curitiba, PR, Brazil; d Secretaria de Saúde do Estado do Paraná, Curitiba, PR, Brazil

**Keywords:** Prevention, Neonatology, Congenital, Latent tuberculosis, Therapeutics, Isoniazid

## Abstract

**Objective:**

To describe the reported cases of newborns subjected to tuberculosis preventive treatment (TPT) in the state of Paraná, Brazil, and to evaluate the safety and effectiveness in preventing the progression of TB disease in this population.

**Method:**

Observational, descriptive case series, with secondary data. The characteristics of the participants were analyzed from the information systems of preventive treatment of TB (of Paraná), between 2009 and 2016. To evaluate which children had developed tuberculosis later or died, we used the data from the information systems of TB (in Brazil), and mortality (in Paraná), covering the years 2009 to 2018.

**Results:**

A total of 24 children underwent TPT with the age at treatment onset ranging from 0 to 87 days (median: 23 days). In 95.8 %, the exposure occurred at home, and in 33.3 % of cases, the mother was the source of the infection. A total of 20.8 % of the children tested positive for tuberculosis test at 3 months of age, 83.3 % completed treatment, and 2 experienced adverse events (gastrointestinal issues). No children developed TB or died during the minimum of a 2-year evaluation period through the official databases.

**Conclusions:**

In this case series, the adherence to the plan was high, with few adverse events and 100 % protection against infection.

## Introduction

Tuberculosis (TB) remains one of the leading causes of death from infectious diseases worldwide. In 2022, 7.5 million new tuberculosis cases were diagnosed (an incidence rate of 133/100,000 population), representing the highest number since the WHO began global monitoring in 1995. Of these new cases, 12 % were children (0 to 14 years old), and TB-HIV co-infection was recorded in 8.5 % of the cases. Brazil is among the priority countries for this disease due to its high burden and co-infection with HIV. In the same period, 80,369 new cases of pulmonary TB were reported (incidence 37.4/100,000 population), with TB-HIV co-infection at 8.5 %. Out of the total new cases, 3.4 % were children (0 to 10 years old), and 0.07 % were neonates.^1^^,^^2^ One of the main tools for TB eradication is prevention.^3^ Regarding newborns, their protection is achieved through tuberculosis preventive treatment (TPT).

TPT aims to treat newborns who are uninfected and unvaccinated but have been exposed to the tuberculosis bacillus. Anti-tuberculosis therapy is administered for a duration of 3 months. Subsequently, a tuberculin skin test (TST) or interferon-gamma test (IGRA) is utilized to ascertain the child's infection status. If the result is positive, treatment continues; if negative, the therapy is halted, and the child receives a BCG vaccination. The duration of treatment depends on the drug used for prevention.^4^^,^^5^

The literature on TB has substantially increased in recent years. However, a significant knowledge gap exists for the newborn age group, as reinforced in the last systematic review in this topic.^6^ The practices in this specific group are often extrapolated from studies conducted in animals or other age groups.^7^^,^^8^ Therefore, this study was designed to describe the reported cases of newborns subjected to TPT in the state of Paraná, located in the southern region of Brazil, and to evaluate the safety and effectiveness of this treatment in preventing TB within the population.

## Materials and methods

### Study design

This is an observational, descriptive case series with secondary data from the TB preventive treatment information systems of the state of Paraná (TILTB database – PR) spanning from 2009 to 2016, the Brazil TB database (TB database – SINAN Brazil) covering the period from 2009 to 2018, and the Paraná mortality information system (SIM – PR) for the years 2009 to 2018. To assess both illness and mortality, the authors analyzed the databases for a minimum of two years following the administration of TPT, as this period would encompass the primary timeframe for the emergence of the observed outcomes.

### Scenario

In Brazil, during the study period from 2009 to 2018, an average of 267 new cases of tuberculosis were recorded in children aged 1 to 365 days, which corresponds to an average of 9.2 new tuberculosis cases per 100,000 live births (LB) among neonates, with an incidence of 1.2 per 100,000 LB. This study was conducted in the state of Paraná, located in the southern region of Brazil, where the average incidence during the study period was 20.3 cases per 100,000 inhabitants across all age groups, 6.5 cases per 100,000 LB among infants under 1 year, and 0.5 cases per 100,000 LB among neonates.^1^

National guidelines for newborns who have been exposed to the tuberculosis bacillus recommend refraining from vaccination and instead initiating TPT, and then performing TST at 3 months. If the TST is positive, they should complete preventive treatment; and if they are negative, therapy should be stopped and the child vaccinated.^5^

### Inclusion criteria

All children under 3 months who were notified in the TILTB database – PR and underwent TPT from 2009 to 2016 were included.

### Procedures and statistical analysis

The characteristics of the participants were analyzed, and subsequently, a comparison was conducted between the two databases to identify children who had developed tuberculosis later. The TILTB – PR database (2009–2016) and TB – SINAN Brazil database (2009–2018) were linked using the OpenReclink™ program, employing the probabilistic record linkage method.^9^ The SIM – PR database (2009–2018) was consulted to ascertain whether any of the children had deceased. The reference variables for data pairing included name, date of birth, and mother's name.

To characterize the study sample, qualitative variables were presented as absolute and relative frequencies, while quantitative variables were described using median, maximum, and minimum values. These statistical calculations were performed using the Stata® program (version 12).

The research was approved by the Human Research Ethics Committee, under approval number CAAE: 9852518.4.0000.5225.

## Results

Throughout the study period, a total of 1617 children and adolescents were recorded in the Paraná state database for TB preventive treatment, among whom 24 individuals (1.65 %) received TPT.

In terms of demographic data, 13 individuals (54.2 %) were male, and 20 (83.3 %) were White. The average age at the initiation of treatment was 30 days (± 5.24; 95 % CI 19.17 to 40.83). All children lived in the urban area and 20.8 % lived in the state capital.

Exposure occurred at home in 23 notifications (95.8 %), whereas in 1 case (4.2 %), it occurred in the hospital. Among the domestic exposures, the identified contact was maternal in 8 cases (33 % of the total); in the remaining domestic exposure cases, the source case was not identified. There was no detailed description of breastfeeding and the type of contact between the index case and the child. No newborns were institutionalized. At the beginning of TPT, 9 newborns (37.5 %) had already received the BCG vaccine.

Regarding the conducted tests, 11 children (45.8 %) had a chest X-ray description not suggestive of TB, and 13 children (54.2 %) did not undergo the examination. HIV testing was performed on only one newborn, and the result was negative. All children underwent the TST, and 5 (20.8 %) showed a reaction of 5 mm or more after three months of TPT. Among them, only one had received BCG vaccination and exhibited a TST induration of 15 mm, indicating infection.

As for treatment, all children received isoniazid, with 9 cases (40.9 %) indicating supervised administration. Concerning the administered doses, 10 cases (45.5 %) received 180 doses, 6 cases (27.3 %) received 90 doses, 2 cases (9.0 %) received 30 doses or fewer, and in 4 cases (18.2 %) the number of doses was unknown. A total of 2 children (11.8 %) experienced gastrointestinal adverse events, and this information was available in 17 cases. No other adverse events were described. The follow-up, described in the database, was the end of TPT in 19 children (79.2 %), 1 (4.1 %) was transferred, and 4 (16.7 %) were unknown.

The details of the cases under evaluation are presented in Table 1.

**Table 1:** Description of the 24 cases evaluated with tuberculosis preventive treatment under 3 months of age, in Paraná – Brazil, 2009 – 2016.

Start of treatment (days)/ Gender/ Race	Source case is mother/Place of exposure/Kind of TB from the source case	Received BCG before investigation	X-ray/ Tuberculin Skin Test	Kind of treatment	Drug used/ Number of doses	Adverse event	Treatment outcome
57, female, white	No, at home, pulmonary bacilliferous	No	No realized, non reactor	Supervised manner	Isoniazid, 90	No	Complete
25, female, white	Yes, at home pulmonary bacilliferous,	No	No realized, non reactor	Supervised manner	Isoniazid, 25	No	No information
79, female, no information	Yes, at home, pulmonary bacilliferous	Yes	Not suggestive of TB, non reactor	Supervised manner	Isoniazid, 180	No	Complete
3, male, white	No, at home, non-bacillary lung	No	Not suggestive of TB, non reactor	Supervised manner	Isoniazid, 90	No information	Complete
81, female, white	Yes, at home, pulmonary bacilliferous	No	Not suggestive of TB, 11 mm	Self-administered	Isoniazid, no information	No	Complete
0, male, white	No, at home, pulmonary bacilliferous	Yes	Not suggestive of TB, 15 mm	Self-administered	Isoniazid, no information	No	Complete
25, female, white	No, at home, pulmonary bacilliferous	No	No realized, 10 mm	Self-administered	Isoniazid, no information	No	Complete
11, male, white	No, at home, pulmonary bacilliferous	No	No realized, non reactor	Self-administered	Isoniazid, 90	No information	Complete
4, male, white	No, at home, pulmonary bacilliferous	No	Not suggestive of TB, 15 mm	Supervised manner	Isoniazid, 180	Gastrointestinal	Complete
16, male, white	Yes, at home, non-bacillary lung	No	No realized, non reactor	Self-administered	Isoniazid, 90	No	Complete
54, male, white	Yes, at home, non-bacillary lung	No	Not suggestive of TB, 5 mm	Self-administered	Isoniazid, no information	No information	Transfer
22, female, brown	Yes, at home, pulmonary bacilliferous	No	Not suggestive of TB, non reactor	Self-administered	Isoniazid, 90	No	Complete
10, female, white	No, at home, pulmonary bacilliferous	No	No realized, non reactor	Self-administered	Isoniazid, 180	Gastrointestinal	Complete
48, male, white	No, at home, pulmonary bacilliferous	Yes	Not suggestive of TB, non reactor	Supervised manner	Isoniazid, 180	No	Complete
19, male, brown	Yes, at home, pulmonary bacilliferous	Yes	No realized, non reactor	Self-administered	Isoniazid, 180	No	Complete
87, male, brown	No, at home, pulmonary bacilliferous	Yes	Not suggestive of TB, non reactor	No information	Isoniazid, no information	No information	No information
36, male, white	No, at home, pulmonary bacilliferous	Yes	No realized, non reactor	Supervised manner	Isoniazid, 180	No	Complete
24, female, white	No, at home, pulmonary bacilliferous	No	No realized, non reactor	Self-administered	Isoniazid, 90	No	Complete
39, female, white	No, at home, pulmonary bacilliferous	Yes	Not suggestive of TB, non reactor	Self-administered	Isoniazid, 180	No	Complete
1, male, white	Yes, at home, non-bacillary lung	No	No realized, non reactor	Self-administered	Isoniazid, 30	No information	No information
12, female, white	No, at home, pulmonary bacilliferous	Yes	Not suggestive of TB, non reactor	Self-administered	Isoniazid, 180	No	Complete
13, male, white	No, at hospital, pulmonary bacilliferous	No	No realized, non reactor	No information	Isoniazid, no information	No information	No information
19, male, white	No, at home, pulmonary bacilliferous	Yes	No realized, non reactor	Supervised manner	Isoniazid, 180	No information	Complete
35, female, white	No, at home, non-bacillary lung	No	No realized, non reactor	Supervised manner	Isoniazid, 180	No	Complete

When comparing the children who underwent TPT with those reported in the Brazil TB database, no new cases of TB were detected among them. Likewise, the SIM – PR database, recorded no deaths within this population. This outcome demonstrates a 100 % rate of protection for the reported cases throughout the minimum 2-year evaluation period, as documented in the official databases.

## Discussion

The percentage of newborns exposed is usually low; however, if they are not identified and protected, the morbimortality of the cases is disastrous due to the high risk of serious diseases, with sequelae or even death.^10^ The present study demonstrated that the majority of contacts were with household bacilli, consistent with the existing literature,^11^ thus heightening the risk for newborns. Nevertheless, even under such circumstances, the likelihood of protection remains substantial provided that appropriate measures are instituted. This underscores the significance of identifying these cases promptly and enacting TPT.

Regarding race/color, it is known that the population most affected by tuberculosis (TB) is Black individuals. However, in this case series, most of the exposed children were White, due to an average of 65.93 % of index cases being White during the same period.^12^ This is further justified by the fact that the majority of the studied population is White, according to IBGE data from the State of Paraná, which indicates a predominance of 64.6 % White individuals in the latest demographic census.^13^

A low frequency of HIV testing was observed in newborns, likely attributable to prior testing undergone by mothers during prenatal care or delivery. Chest radiography was not performed in a little more than half of the patients, as this examination should only be done if the child is symptomatic or if the index case is the mother, in order to rule out congenital TB.

Regarding the doses taken, 79 % of the cases were considered to have adequate regimens, with 180 doses or 90 doses (in those who had a non-reactive TST after 3 months). For the remaining cases, the authors cannot attribute the absence of illness to the treatment. However, these cases were important to demonstrate the safety of the medication, as no adverse events were reported.

After the treatment, the majority of children exhibited non-reactive TST, signifying the protective efficacy of the treatment in preventing the infection. The authors encountered a lack of comparable studies in the literature for data comparison.

In the present study, all children received Isoniazid, the primary regimen recommended during that period. Only two children experienced adverse events. Although studies in this age range are scarce, the safety profile of drugs employed in TPT appears to be similar to other age ranges.^7^^,^^14^ Notably, although the use of pyridoxine in conjunction with isoniazid, as recommended by WHO,^4^ was not employed, and no instances of neuropathy were documented. Since this is a study with secondary data, the safety analysis may have been compromised by possible underreporting of these events, thereby constituting a study limitation.

The compliance with the prescribed regimen exhibited a notable degree of adherence, suggesting that the principal impediment to this preventive approach possibly resides in its endorsement by the healthcare personnel.^15^ This observational study of real-world scenarios distinctly showcased the safety of TPT within the neonatal age range, thereby reinforcing observations previously documented in clinical trials involving alternative age cohorts.^16^

This study had limitations inherent to its reliance on secondary data from notification databases. It did not involve direct patient follow-up, and certain data proved challenging to interpret, including the confirmation of the effectiveness of the preventive treatment.

The current guidelines concerning the management of newborns born to mothers with tuberculosis exhibit heterogeneity across various countries and are marked by substantial lacunae in both knowledge and clinical practice. In this case study, there was notable adherence to the outlined plan, characterized by minimal occurrence of adverse events and a remarkable 100 % protection efficacy against infection.

New prospective studies should be encouraged in this age range to ensure the safety and protection of this population.

## Conflicts of interest

The authors declare no conflicts of interest.
